# Contribution of participation and resilience to quality of life among persons living with stroke in Sweden: a qualitative study

**DOI:** 10.1080/17482631.2022.2119676

**Published:** 2022-09-04

**Authors:** Marie Matérne, Grahame Simpson, Gustav Jarl, Peter Appelros, Mialinn Arvidsson-Lindvall

**Affiliations:** a University Health Care Research Center, Faculty of Medicine and Health, Örebro University, Örebro, Sweden; b School of Law, Psychology and Social Work, Örebro University, Örebro, Sweden; c John Walsh Centre of Rehabilitation Research, University of Sydney, Sydney, Australia; d Brain Injury Rehabilitation Research Group, Ingham Institute for Applied Medical Research, Sydney, Australia; e Department of Prosthetics and Orthotics, Faculty of Medicine and Health, Örebro University, Örebro, Sweden

**Keywords:** Content analysis, health, interview, rehabilitation

## Abstract

**Purpose:**

Resilience contributes to positive adaptation after many health conditions, but little is known about its contribution to long-term recovery after stroke. This study investigated the lived experience of resilience and participation and their relationship to quality of life after stroke in Sweden.

**Material and method:**

Semi-structured telephone interviews were conducted with 19 informants (10 male, 9 female), aged from 44–89 years and between 1 and 19 years post-stroke. Stroke severity ranged from mild (n = 8), moderate (n = 9) to severe (n = 2). Interviews were analysed using content analysis.

**Results:**

The analysis resulted in an overarching theme; ***Life with stroke has been adapted to but not accepted***, built on five subthemes: 1) Adapting and adjusting life, 2) Meaningful values in life, 3) Inner resources, 4) Support and treatment from social relations, and 5) Support and treatment from external resources.

**Conclusion:**

Participants described a tension between adapting and accepting life after stroke. Resilience was a useful framework, highlighting the contribution of inner, social and societal resources to recovery and quality of life, both directly and as enhanced through increased participation. Important factors for adaptation are meaningful values in life, individual strategies for adaptation and support from both social relationship and the society.

## Introduction

Stroke is a major contributor to the global burden of disease (Feigin et al., 2003; Mukherjee & Patil, 2011) and to disability within Sweden (Appelros, Arvidsson-Lindvall et al., 2021; Riks-stroke, 2019). The World health Organization, defines stroke as ”rapidly developing clinical symptoms and/or focal, and at times global, loss of cerebral function, with symptoms lasting more than 24 hours or leading to death, and no apparent cause other than of vascular origin” (Aho et al., 1980). The onset of stroke poses a life-threatening challenge in the short term, followed by a period of functional recovery, often associated with rehabilitation. Within Sweden, the lived experience of stroke during this acute phase has been documented in a small number of studies, focusing on impairments such as pain (Widar et al., 2004) and balance (Arvidsson Lindvall et al., 2021), alongside the broader psychological and social challenges of adaptation, characterized as living with struggle and uncertainty (Carlsson et al., 2009) or a desire for belonging/integration (Eriksson et al., 2009). However, less is known about the lived experience of stroke and the adjustment challenges in the chronic long-term phase. There is also a need for more knowledge within a Swedish context due to differences in culture compared to other countries. For example, Sweden is a strongly secular society, with religion playing a lesser role compared to many other European countries as well as North America. Moreover, at a societal level, most human service support systems are publicly funded with universal access, compared to countries in which services are predominantly delivered on a selective basis through the private sector.

The Kumla stroke study provided the opportunity to address this knowledge gap. Kumla is a small municipality (popn approx. 23,000) located in Sweden. The study identified the entire population of persons with stroke (n = 330; 1–37 years post-stroke) living in this municipality (Appelros, Arvidsson-Lindvall et al., 2021). Few studies with a focus on lived experience have been able to both include people at long time-points post-stroke and sample systematically from a single jurisdiction (Sarre et al., 2013).

In the broader literature, quality of life (QoL; World Health Organization Quality of Life Group, 1993) is a core goal in stroke rehabilitation, seeking to achieve the best outcomes possible (Wiklund, 2004) in the context of physical, cognitive, psychological and social challenges (Wolfe et al., 2011). In understanding the adjustment process underpinning QoL, a wide range of individual, relational and structural factors have been identified in previous qualitative studies (Sarre et al., 2013). Two interrelated constructs of resilience and participation may be useful conceptual frameworks that encapsulate many of such factors reported in these previous studies.

In their 2014 review, Sarre and colleagues highlighted the growing research attention focusing on the role of resilience in adaptation to a range of health conditions (Sarre et al., 2013). Conceptually, it encompasses a range of different thoughts, feelings and behaviours that individuals use to handle and adapt to adversity, and this has been explored within the rehabilitation context (Luthar et al., 2000; White et al., 2008). Resilience is posited to be a multi-dimensional construct comprising a mix of personal skills and attributes, social competence, social resources and spirituality, which may be associated with reductions in morbidity and increased positive wellbeing (White et al., 2008). Despite this promise, Sarre et al. found there was limited research within stroke that had examined this psychological resource (Sarre et al., 2013).

Initial studies have identified that resilience makes an independent contribution to QoL after stroke (Liu et al., 2019; Zhou et al., 2020). In the acute setting, higher resilience was found to be negatively correlated to anxiety and depression (Liu et al., 2019). It was also associated with higher quality of life, independent of socio-demographic, disease-related, physical functioning and mood-related variables (Liu et al., 2019). The authors concluded that resilience both enhanced QoL and acted as a buffer against anxiety and depression post-stroke, but stated that research that extended into the post-hospitalization phase was needed.

Empirically, quantitative studies have reported an association between participation and resilience (Ponsford et al., 2014) after traumatic brain injury (TBI), and a similar relationship may also be found after stroke. Participation focuses on the involvement in a life situation (World Health Organization, 2001) and this encompasses broad domains such as work, leisure, mobility (inside and outside the home), relationships and other aspects of community life. Many such activities have been described as ‘identity-defining” (Clarke & Black, 2005; Hartke et al., 2011) and provide strong social connections (Eriksson et al., 2009). It includes the sense of re-integration into society (Eriksson et al., 2009) and these domains of participation would seem to overlap with resilience. However, little is known about the lived experiences of these domains after stroke, and their association with QoL. Furthermore, QoL is sensitive to, and varies between different cultural contexts (Ayis et al., 2015; Sprigg et al., 2012). Therefore, the aim of this study was to explore the experience of the contribution of participation, and resilience to QoL among people who have had a stroke in Sweden.

## Materials and methods

This study was approved by the Swedish Ethical Review Authority (reference No. 2019–02359). All participants were given written and oral information about the study and gave written consent to participate before the interview. In addition, the paper has followed the Consolidated criteria for Reporting Qualitative research (COREQ; Tong et al., 2007).

Informants were recruited from the Kumla stroke study. Participants in the larger prevalence study were identified through the Swedish stroke register and their diagnosis was confirmed in the medical records (Appelros, Arvidsson-Lindvall et al., 2021). A total of 281 (85%) had an ischaemic stroke, 35 (11%) an intracerebral haemorrhage and 12 (4%) a subarachnoidal haemorrhage, in total 330. The majority (58%) were men and the mean age was 74.5 years (SD 13.7). Thirty-eight (11.5%) had more than one strokes. The mean duration since they had their first stroke was 8.3 (SD 6.3) years and comorbidities were common. For example, 80% had hypertension, 30% orthopaedic disorders, 18% ischaemic heart disease, and 12% had cognitive impairment (Appelros, Matérne et al., 2021).

### Informants

The main inclusion criteria were (i) resident in the Kumla municipality on the 31th of December 2019, (ii) having had a stroke and (iii) fluent in speaking Swedish. A total of 330 participants with stroke were identified in the Kumla stroke study (Appelros, Arvidsson-Lindvall et al., 2021). From this, a purposive sample was chosen by the researchers MM and MLA, aiming for heterogeneity (Patton, 2002) regarding age, sex (male, female), occupation (retirement pension, sickness compensation, different occupations) and severity of the stroke (mild, moderate, severe). Another inclusion criterion was to be. The exclusion criteria comprised having severe aphasia or other extensive communication problem. Given the broad timespan post-stroke among the sample, the severity of the stroke was self-reported (see, Table 1) and confirmed by the research team.

**Table 1.** Characteristics of the informants at the time of the interview.

Sex	Age	Occupation	Marital status	Years since stroke	Residual symptoms	Severity of stroke	Self-reported problems after stroke ^a^	Self-reported return to activity ^b^
M	89	RP	P	4	Half-side paralysis, fatigue, memory loss	Moderate	2	3
F	81	RP	S	9	Numbness left hand	Mild	2	1
M	80	RP	S	6	Aphasia	Moderate	2	2
M	75	RP	P	4	Fatigue, dizziness, reduced balance, loss of sensation	Mild	2	2
F	75	RP	P	4	Memory loss	Mild	1	1
F	74	RP	P	5	Memory loss, slightly lameness	Moderate	2	2
M	70	RP	P	2	Half-side paralysis, memory loss	Moderate	2	2
F	69	RP	P	3	Fatigue, dizziness and left foot paralysis	Moderate	2	3
F	60	SC	S	8	Spasticity, can walk with a stick, wheelchair user	Moderate	2	2
M	60	SC	P	11	Left side paralysis, spasticity, can walk	Moderate	2	2
F	58	Part time assistant at school, part time SC	P	6	Fatigue, memory loss and aphasia	Severe	2	3
M	57	Transport manager	P	3	Right side paralysis, memory loss	Moderate	2	1
M	57	Finance assistant	P	19	Balance, dizziness, fatigue, memory loss and mood swings	Mild	2	2
F	55	Human resource manager	S	2	Numbness right side	Moderate	3	2
M	54	Teacher	P	13	Balance problem and fatigue	Mild	2	1
F	46	Teacher	S	19	Right side paralysis	Moderate	3	2
M	45	Work training—industry	S	1	Double vision	Mild	2	2
F	44	Administration	P	2	Right side paralysis and fatigue	Mild	1	1
M	44	SC	S	1	Left side paralysis, wheelchair user and fatigue	Severe	2	3

RP = retirement pension, SC = sickness compensation, S = single, P = partner. ^a^ Question from the Stroke questionnaire, rating scale: 1 = All the problems have passed completely, 2 = I still have problems 3 = I do not know. ^b^ Question from the Stroke questionnaire, rating scale: 1 = Yes, 2 = Yes, but not quite as before, 3 = No, 4 = I do not know.

Twenty-six potential informants were contacted by telephone, informed about the study and invited to take part. Five persons declined to participate: two due to lack of expereine of stroke symptoms, one due to aphasia that prevented participation in a telephone interview and two declined without giving a reason. In addition, two persons gave initial consent to participate but could not then be reached by telephone to book the interview. Thus, 19 interviews were conducted. No relationships between the informants and the interviewers were ongoing during or under the interviews.

### Procedures

The study comprised a cross-sectional qualitative design. To enable the informants to talk freely, a semi-structured interview was conducted (Richards & Morse, 2013). The interview guide drew upon concepts of participation embedded within the International Classification of Functioning, Disability and Health (ICF) model (World Health Organization, 2001) and resilience from the previous theoretical work of White and colleagues (White et al., 2008). The interview guide was piloted with one stroke survivor, not included, before the study started. As the participant understood all questions, no changes were made to the interview guide.

The interview guide consisted of four key areas; 1) background characteristics, 2) QoL, 3) participation, and 4) resilience (see appendix 1). Each interview lasted for about 30–45 minutes and were done one by one. Twelve interviews were conducted by MM (female), a registered health care counsellor and researcher (PhD) with more than 25 years of experience of working with people with disabilities. Seven of the interviews were conducted by a social worker (MsC, female) at an adult habilitation centre with 5.5 years of experience working with people with stroke.

Within the research group regular meetings took place to review the data, and after 19 interviews, data saturation had occurred with no new themes emerging from the interviews and therefore no further informants were recruited (Kvale & Brinkmann, 2009; Sandelowski, 1993).

### Analysis

All interviews were audio-recorded and transcribed verbatim. Qualitative content analysis was used to captures variations in the material (Graneheim & Lundman, 2004). The data was structured using the qualitative software programme NVivo 11 (QSR International, Inc., Cambridge, MA, USA) to support the analysis. Two of the authors (MM and MLA) read and reread the interviews several times to become familiar with the data. The analysis then was conducted by an inductive approach with focus on answering the aim, starting with coding on a manifest level with no interpretation of the material and then continuing with a superior interpretation and a more latent level (Graneheim & Lundman, 2004). The steps in the analysis were inspired by Graneheim and Lundman (Graneheim & Lundman, 2004), and of five analytic steps: meaning units, codes, categories, subthemes and overarching theme. In this study, we excluded a step with condensed meaning units due to the informants often brief answers, even to follow-up questions, which means that the meaning units did not have to be condensed. Subthemes and one overarching theme were used instead of only themes. The first two steps with meaning units and codes were related to the manifest content, while the last steps with categories, subtheme and overarching theme, were made through interpretations of a more latent level. The first author, (MM) analysed the manifest level with meaning units and codes, discussed the analysis with another researcher (MLA). On the more latent level, the whole research group then discussed and revised the categories. Finally, each researcher independently formulated subthemes and overarching theme, which were then discussed in the research group until consensus was reached (Richards & Morse, 2013). This was a way of seeking trustworthiness of the material (Hsieh & Shannon, 2005).

To clarify validity through the convergence of information from different data sources we used both qualitative and quantitative methods. The main data collection were through telephone interviews and additional data were on participants’ demographic characteristics, including age, gender, place of residence, living with a partner or alone, this data were collected before the interview started and from earlier datacollection (Appelros, Matérne et al., 2021). At the end of the telephone interview the participants were encouraged to give further information if needed.

## Results

Nineteen informants (ten men, nine women) were included in the study (see, Table 1). The informants were aged between 44 and 89 years and their stroke occurred between 1–19 years previously. Stroke severity ranged from mild (n = 7), moderate (n = 10) to severe (n = 2). Eight informants were on retirement pensions, eight had returned to work full or part time and three were receiving full time sickness compensation. Several of the participants reported residual functional impairments across physical (hemiparesis, spasticity, loss of balance) and cognitive (dizziness, fatigue, memory loss) domains, see, Table 1.

The overarching theme was ***Life with Stroke has been adapted to but not accepted.*** All informants described situations that illustrated how the stroke had affected their life and how they had adapted their life due to the stroke. Almost everyone had found ways to cope with the life after stroke but they did not accept the situation. The lack of acceptance was bidirectional. For some, there was a resignation that their lives had changed permanently, and this had to be accommodated. Others however, were still working to overcome many of the limitations and challenges that they faced as a result of the stroke. This overarching theme was built on five subthemes: 1) Adapting and adjusting to life, 2) Meaningful values in life, 3) Inner resources, 4) Support and treatment from social relations, and 5) Support and treatment from external resources. These five sub-themes are described below with citations from the informants, see, Table 2.

**Table 2.** Findings divided into categories, subthemes, and overarching theme.

Categories	Subthemes	Overarching Theme
1. Accessibility and adaptation to a life with stroke 2. Leisure time is different but still active after the stroke 3. Consequences of the stroke affect everyday life	Adapting and adjusting to life	Life with Stroke has been adapted to but not accepted
1. Spirituality and faith create meaning 2. Quality and values in life are different 3. The importance of family and closest friends	Meaningful values in life	
1. Persistence, motivation and problem solving creates independence 2. Satisfied with the recovery and acceptance with oneself	Inner resources	
1. The feeling of being useful 2. Social relationships work well and have sometimes deepened 3. Social relationships have become complicated and how they were treated	Support and treatment from social relations	
1. The home service is careless and changes staff all the time 2. Lack of follow-up from health-care and society 3. Assistive technology, assistance and support increase accessibility and facilitate activities	Support and treatment from external resources	

## Adapting and adjusting to life

This first subtheme was about how the informants were adapting and adjusting their life due to their new, often suddenly occurring, situation. All informants described that the stroke had been dramatic. However, the stroke had changed life dramatically for some informants, but had no long-lasting effects on other informants. In this subtheme, three categories were identified: 1) Accessibility and adaptation to a life with stroke, 2) Leisure time is different but still active after the stroke, and 3) Consequences of the stroke affect everyday life.

In relation to the category of ***accessibility and adaptation to life***, almost every informant described the varying degrees of adjustment to stroke that were needed. Several of the informants with physical disability reported that the accessibility had become more difficult and they were dependent on their physical environment and assistive devices due to physical impairments. For example, a 70-year-old man who used a mobility scooter faced the challenges/barriers associated with the amount of space needed to store the scooter, as well as the problem of still not being able to access all places due to physical barriers such as stairs.

*Yes, in most places it works. Ehh … it is … of course you choose places now that I, you know … that you can access. (M/70 years)*

His life had changed a lot after his stroke and he had adjusted to limitations in where he could go, based on the accessibility of the desired destination. For example, it affected which friends or family he could physically visit due to his extensive disabilities in movement.

On the other hand, mild symptoms also restricted participation in life. A young women described letting her husband take more responsibility for driving their teenage daughter to training at late hours due to fatigue.

*Yes. No huge difference. But I have, or we have a daughter who exercises quite a lot, has late-night workouts, obviously I haven’t picked her up in the last year [due to my stroke]. (F/44 years)*

The informants described that ***leisure time was different but still active***. An old man, severely affected by balance difficulties and dizziness, described how he and his wife tried to live their life and spend their leisure time as before he was injured. He took more pauses to compensate for the injury, but he still thought of his life as meaningful and active.

*Yes . that’s it … it’s exactly the same things, we try it … to … continue in the way that has energized us and given us meaning before, as far as we can. (M/ 75 years)*

A middle aged woman recounted how her life and leisure time had changed. She had made changes in some of her former leisure activities, and could manage due to her determination, while others activities were impossible to do. She felt uncomfortable not managing her leisure time herself anymore, which gave rise to feelings of inadequacy which she had to deal with.

*No I have not. Or rather … I try to do what I can do, but naturally I have realized that some things cannot be done, e.g., go swimming and stuff like that [giggles]. And of course it isn’t usually swimming with my children either, but my husband then had to do that, but there you go. Otherwise, I try to do as much as I can, because I am very determined, I generally don’t want help, I want to manage things myself. (F/ 46 years)*

The informants described how ***consequences of the stroke affect everyday life*** in different ways considering their disabilities arising from the stroke. Difficulties with speaking, reading and writing due to aphasia were one consequence experienced as affecting everyday life

*And that … and a lot, read a lot and wrote a lot and talked a lot [laughs] all of that, you have to put that aside now. (M/ 80 years)*

Several of the informants described functional impairments affecting them in everyday life. Substantial walking difficulties due to dizziness and balance problems were some consequences. Fatigue and mood changes were others. The consequences of the stroke meant that they had to make adjustments to their lifestyle compared to their lives before the stroke. A young woman described how her fatigue affected her social life.

*I have probably never been one of those who liked to be out partying and so on, but of course I can meet friends and all that on the weekends, but now I basically just want to be home and have a good rest, quite simply. And I think that “Yes but okay, if I meet her on Saturday, or my friend on Saturday, then I know I’ll feel bad on Sunday.” (F/ 46 years)*

## Meaningful values in life

The second subtheme was about values in life, conducive to QoL. For some of the informants, life did go on almost like before but for others values in life had dramatically changed. The subtheme had three categories: 1) Spiritualty and faith create meaning for some but had no value for others, 2) Quality and values in life are different, and 3) The importance of the family and the closest friends.

Some informants talked about how ***spirituality and faith created meaning*** but others said that ***spirituality did not create added value.*** Most of the informants did not find any comfort in spirituality neither before nor after the stroke. The church had contributed to practical support as with money and procurement which one man experienced as valuable. He had also found people to talk with from the church, but he did not find any comfort in the faith. Another man who had been a Christian his whole life said that he had benefitted from the comfort and hope that he found in his faith even after the stroke.

*Yes, to be sure it has been, so to speak … a power, a strength for me, a meaning throughout my life, you could say, so it has not meant any change here but it has rather been I feel a deep faith although things happen that you don’t want to happen, so I have still seen felt the strength to move on, not to give up at any time, I haven’t felt that way. (M/ 76 years)*

The informants described that ***quality and values in life are different*** after they got their stroke. Several experienced a change in their QoL, either in a positive or a negative direction. One women explained how she now had a greater gratitude to life than before her stroke. She woke up every day with a smile on her face.

*I have kind of, I appreciate life more, I must say and it is probably not a disadvantage but you used to take everything for granted, I think. But now I think every day is quite wonderful, really [laughs]. But I look at it so differently before and after, actually. (*F/ 69 years)

On the other hand, several of the informants experienced a decline in their QoL by not being able to do the things they wanted anymore. One informant explained that his life completely had changed and he felt depressed. His friends did not understand his situation and how hard it was to struggle with the disability.

*No. I’m feeling terrible, I’m getting depressed, I’m depressed right now. I’m just lying in bed and not doing anything. It feels like they [i.e., friends] have distanced themselves, I don’t think they really understand how I feel, how hard it is with a stroke. (M/ 44 years)*

The last category in this theme was about ***the importance of family and closest friends.*** Almost all participants highlighted an increased appreciation of the importance of those who they were closest to in their social networks. They thought that the value of the support provided through their social network was crucial to their QoL and their continued life after the stroke.

*Yes, I have had support from my husband, I must say. And so it’s probably the support that maybe—I may not have felt it, or not needed any other support, you could say, from outside, of others, girlfriends and so on to some extent, but like I said, it’s my husband who is my support. (F/ 75 years)*

For the informants who had children it was hard to continue being a parent in the same way as before. A man who had separated from his partner just before he got the stroke felt very sad. However, he described the value of his daughter who gave him a meaning to continue living his life.

*She stayed [when we separated] and I moved to [the town’s name] and then only a month went by and then this happened [the stroke]. So I have my daughter every other week. This has helped me a lot to come back. When I was paralysed and couldn’t talk and eat, I had that to fight for, it helped me a lot mentally. We are very tight, it’s daddy’s girl, she prefers to be with me all the time. And then I just disappeared and she, unfortunately she was with me when it happened. (M/ 45 years)*

## Inner resources

This theme focused on each person’s own resources. It was about their motivation level, how they could solve problems that appeared and how they felt about themselves and their personality. Two categories were identified: 1) Persistence, motivation and problem solving create independence, and 2) Satisfied with the recovery and acceptance with oneself.

For many of the participants, inner resources such as ***persistence, motivation and problem solving created independence*** and were therefore important in their recovery from the stroke. One man described how he had solved problems for himself and others during his whole life and continued with that even after the stroke. It created a value in life for him to be a problem solver and he found a meaning with life thanks to his skills within this area.

*Yes, but I have solved problems for both myself and others throughout my life. (M/ 80 years)*

Another man who also was a problem solver found that when he got tired he could rest for a while and this would refresh his cognitive functioning.

*Because I … feel that … to a very large extent, my thinking still works of course and then and I … I have a bit of that inventiveness and find solutions for most things. So that the moments when … my mind has gone blank here, then I go to bed and rest until it works again, and then I don’t solve any problems at that very moment. But it is still such short moments that it is negligible, I think. (M/ 75 years)*

The second category was about ***the satisfaction with the recovery and tolerance with oneself.*** An old woman who had stroke nine years ago found that, as she did not get any help from others, she had to trust her own power and self-reliance.

*My strength is within myself. I have realized that you can’t shift it on to some spirituality but [laughs] no, you must have power over yourself. I don’t know if that’s the right answer, or a good answer, but that’s the way it is. (F /81 years)*

Several informants talked about seeking to have a balance between different needs such as a social life, love and economic resources. For one man this balance was experienced as still being able to work as a teacher even after the stroke. He thought that having a decent income for both his and his partner’s security was important.

*It means a … ehh yes a reality and an experience of … having a … a certain independence, not completely independent, but that … your existence that the basic things in life are met and that it is in an even balance with what you want, socializing, you want security, love, closeness. And to achieve that, it usually requires in … at least as I have experienced life so far that you have some form of income that can both be maximized and can be high, but so it is sufficient to fulfill it, that is, a job or other income. (M/54 years)*

## Support and treatment from social relations

The informants described the importance of the social environment, including support and treatment by others. To feel useful, have deepened social relationships and be treated as before the stroke increased their feelings of value. Three categories emerged from the interview material: 1) The feeling of being useful, 2) Social relationships work well and have sometimes deepened, and 3) Social relationships have become complicated and how they were treated.

The first category was about ***the feeling of being useful.*** Several informants described that it was important to mean something for others, for their children and, for some, their grandchildren. To have a value was experienced by one informant, living alone when he got his stroke, was in receiving an offer from his friends to come and live with them and be supported while undergoing his rehabilitation. This offer of support and friendship made him felt valuable and that he meant something to someone else.

On the other side, to feel that you do not have any value in life was depressing and devastating. One man described how he could not do the things he did before with his son and as a result, felt he had no value to anyone nor to himself any more.

*Then I have feelings of guilt because I have an illness and can’t do things with my son, as I did before, was out cycling and we were out on very long, long distances etc., etc. No so no, I think stroke is terrible. (M/ 44 years)*

For some informants ***social relationships worked well and sometimes deepened*** and they did not experience any change in how people treated them compared to before the stroke. One man described that both his family and friends had accepted and treated him as before the stroke despite the impact the stroke had on his cognitive ability and fatigue.

*It is hard to say. For example, within my family, it’s what … it is as it was before, but friends have also adapted and the friends I had before [my] stroke and I also have them now and I also have contact with them. (M/ 57 years)*

In contrast, several of the informants described that their friends and ***social relationships had become complicated and how they were treated***. They described that their friends had distanced themselves. One man with aphasia felt that losing part of his possibilities to express himself also affected the possibilities to make contact that was meaningful, so that making new social relationship was no longer a joy for him.

*Because if you can’t talk and get across what you want to say and feelings and everything like that, then … it means that you have a hard time making new contacts of a meaningful nature. (M/ 80 years)*

A woman living with dizziness, fatigue and a hemiparesis after her stroke, who also had cancer, described that her friends distanced themselves from her and her husband. She thought they were afraid.

*And it’s true, even our friends have … although the friends you can also see a little sometimes that they may distance themselves a bit because they are afraid of “Yes, what should you say and so on” because that’s how I am too [laughs] if you maybe, yes get sick and hard to get into the life of a friend if one says, so then I can … yes … it has been a bit like that … you have … Because it was not just that I got sick but my husband—it actually started with my husband getting cancer in his stomach … it was 2015 and that was where it all started. (F/ 69 years)*

## Support and treatment from external resources

The last subtheme was about the external support and treatment. The support and treatment from external resources was built on national rules and regulations. However, the treatment from the professionals could not be regulated and the informants felt differently about how they were treated by the society and health care. The informants described the support and treatment in three categories; 1) The home service is careless and changes staff all the time, 2) Lack of follow-up from health-care and society, and 3) Assistive technology, assistance and support increase accessibility and facilitate activities.

The first category was about ***the home service is careless and changes staff all the time.*** One man with extensive need for assistance felt that the home service was unprofessional in their treatment of him and could not fulfill his needs for help. He did not trust them and therefore did not want the help from them despite his extensive need for help.

*And that’s the way it was, I have a hygiene qualification so I saw [that], then I get stressed. I got close … close … to being poisoned too / … / I got a double dose of the medicine so I was poisoned by that as well one day, so I was nearly … nearly poisoned there, I got a low pulse. But … no the home care service has been terrible, they have come at odd times of the day, no routines really, it has made me stressed. That’s why I declined all the time so I get peace and quiet, it’s only in the morning that they come. (M/ 44 years)*

The other category within this theme was about ***lack of follow-up from healthcare and society.*** Several of the informants felt that they did not get the support and help from society and healthcare that they needed or were entitled to. A man with a hemiplegia felt that he had needed more rehabilitation in connection with his stroke.

*No, so you would of course like more support from the hospital. To not just be discharged and then that’s it. It is bad follow-up so to speak. (M/ 60 years)*

The last category in this theme was about how ***assistive technology, assistance and support had increased accessibility and facilitated activities*** for the informants. One woman who had a right side paralysis received financial support and practical help to adapt her car. That helped her to live an active life with work and children.

*No, but I can cope. I have a car with that kind of handicap- or what is it called, handicap adaptation, with one of those steering wheel balls on, so I can drive. And I can manage to park, I don’t park on the disabled parking spaces but I … can manage it that way, to be sure. But it is clear that I can’t walk longer distances, of course it isn’t possible. But otherwise … I think it works pretty well, I’m determined as I said (laughs). (F/ 46 years)*

Another informant described how happy he was with the support he got from the society, the rehabilitation and the employer. He thought that without them he maybe couldn´t have returned to work again after his stroke.

*I had support from ehh … employer, social insurance agency and employment agency and that helped me a lot that I have come back. Not like a hundred but like fifty percent, that much anyway, that’s good. (M/ 57 years)*

## Discussion

The overarching theme of the study was *Life with stroke has been adapted to but not accepted*. Many aspects of this process of recovery/adjustment could be conceptualized as fitting under a twin framework of resilience and participation. Many of the findings from the results can be integrated and interpreted through a resilience lens including the domains of meaning-making, as well as inner, social and societal resources. Participation has also been linked to resilience after traumatic brain injury (Wardlaw et al., 2018) and the current study suggests that similar associations can be found in the stroke.

The results painted a picture that living with stroke over the long-term was not a static plateau but a dynamic process where different resources are used to support adaptation and acceptance to improve QoL. However, this process was not all smooth flowing, as there could be limitations, setbacks or failures in the resources at each three levels (inner, social and societal resources) which created barriers and challenges to the adjustment process. The dynamic nature of the process over the long-term reflected findings from a recent Australian stroke study, in which the main theme of *Living my life, as it evolves* was elicited (Jackson et al., 2021).

The concept of “adapted but not accepted” was either positive or negative. Starting with “not accepted”, for some, the lack of acceptance was a driver motivating work towards recovery, consistent with previous reports (e.g., (Kouwenhoven et al., 2011)). For others, the lack of acceptance was associated with a sense of “resignation”, reflecting a less positive appraisal of the life change associated with stroke (Sarre et al., 2013), and this can further extend to post-stroke depression (Robinson & Jorge, 2015). Alternatively, for the informants who had come to an acceptance post-stroke, this was associated with more positive emotions, which is generally associated with better mental health and QoL (Ch’ng et al., 2008; Clarke & Black, 2005), highlighting the importance of positive emotion. In other neurological (individual or carer) samples, resilience was strongly associated with positive emotion which contributed to positive mental health and acted as a buffer against negative affect and psychological distress (Simpson et al., 2021).

Meaningful activity was an expression of participation, contributing to both QoL and resilience. The impact of stroke on participation reflected findings from previous studies (Clarke & Black, 2005; Hartke et al., 2011), with the adjustments made to preserve valued activities such as leisure, work and community mobility associated with significant psychological, social and financial benefits (Clarke & Black, 2005; Hartke et al., 2011). Many of these elements of participation also reflect domains of resilience relating to social integration and access to resources (Sarre et al., 2013). The findings highlighted the importance of participation, in the form of meaningful activity to overall QoL (Clarke & Black, 2005; Eilertsen et al., 2010; Wood et al., 2010).

The role of meaning-making is an important element in the adjustment process (Sarre et al., 2013). Some participants in the study drew upon their spirituality, consistent with previous reports (Cross & Schneider, 2010; Gibbs et al., 2020). Studies in other neurodisability groups have also found a strong association between spirituality and resilience (Jones et al., 2019; Simpson et al., 2020) and similar relationships may exist after stroke. However, only a few participants in the current study identified spirituality as a source of strength, with the majority not reporting that spirituality played any role in their adjustment, reflecting the secular nature of Sweden (World Values Survey 7, 2020), so it may not be as interrelated with spirituality within the Swedish context. In the current study, self-reliance was understood as an important component of resilience (Wagnild & Collins, 2009) and this may provide a secular alternative to spirituality. This then reflects cross-cultural differences in the nature of resilience and how it might contribute to QoL.

Inner resources are well understood as part of resilience (White et al., 2008) and can include gratitude, either understood as part of spirituality (Manning, 2014) or independent of spirituality (Chun & Lee, 2013). The reporting of gratitude as a response to stroke, is consistent with previous reports of gratitude from other neurological groups (Jones et al., 2018). Little research to date has been conducted into the field of gratitude in the context of health conditions (Elosúa, 2015) and our findings highlight this as an avenue for future research in stroke.

Other types of inner resources reported by participants such as perseverance (Brown et al., 2010) have been reported in previous stroke studies, however problem-solving (as a more specific cognitive mechanism in contrast to problem-focused coping) has not been widely studied to date. In caregivers of people with TBI, problem solving and self-efficacy were both strongly associated with resilience and hope (Anderson et al., 2020), and a similar relationship was found in this study among people with stroke. Finally, participants also related stories of struggle with depression, a common consequence of stroke (Robinson & Jorge, 2015). Of interest, studies both among stroke patients (Zhou et al., 2020) and other neurological groups (Jones et al., 2019) have found that resilience can be a buffer to depression, although further longitudinal studies are needed to support these early findings.

Resilience is not simply a matter of inner resources but also relates to the ability to mobilize social and economic resources to make the challenges of living and adapting to life challenges (viz. stroke) manageable (White et al., 2008). This highlights another intersection between resilience and participation, as informants who were able to return to work found that it contributed to their psychological, social and economic well-being. In terms of family and social networks, the feelings of being valued by others reported by informants reflect findings in previous reports (Bays, 2001; Erikson et al., 2010), and families were one avenue for accessing resources for the person with stroke (Ch’ng et al., 2008; Lynch et al., 2008). However, participants also identified challenges in relation to relatives distancing themselves, or family and friends not understanding the impact of stroke on an individual (Brown et al., 2010; Murray & Harrison, 2004), and these types of reactions can pose a threat to the development of resilient responses to the challenges posed by stroke.

External resources, which include support at a societal level that contributes to individual and social levels of resilience, has received limited attention in the literature to date (Sarre et al., 2013). Participants’ experience of poor services from home support providers and/or the feeling of abandonment arising from the abrupt discharge from health services and lack of follow-up have also been previously documented (Brown et al., 2010; Murray & Harrison, 2004), and present a real threat to individual resilience. This was further exacerbated by the impact of physical barriers that reduced community participation and access to social supports (Murray & Harrison, 2004). In contrast, good services increased accessibility and facilitate activities (F Jones et al., 2008; Murray & Harrison, 2004), enhancing both participation and QoL. The financial impact of stroke can be considerable (Koch et al., 2005; McKevitt et al., 2004) and the benefits of work enabling income contributed to the resources available to the person with stroke and played an important role in supporting QoL within this sample.

## Limitations

The study had a number of limitations. Although the sample were recruited from a single jurisdiction, there is no guarantee that the themes elicited from the participants are representative of the key concerns of the broader stroke population in Kumla. Next, the study was conducted during the corona virus pandemic, and therefore the interviews were conducted by telephone rather than face-to-face, which may have led to a loss of interview quality and/or reliability. Other techniques for interview for example, video conference could have been used, a secure video-conferencing platform was not available, and informants having suitable technology to receive such calls was not a selection criterion for the study. Phone interviews provided a familiar and “easy to use” method for the informants to participate in the interview. Furthermore, telephone interviews have been shown to be a valid method for data collection (Sturges & Hanrahan, 2004).

It may seem curious that the informants talked so little about physical impairments, which are common after stroke (Langhorne et al., 2009; Pollock et al., 2014). This was due to the focus of this study, and thus, impairments were given less attention although they were common in the sample (Table 1). Therefore the full role that such impairments might play in relation to participation and resilience may not have been fully elicited. Finally, the informant group had their stroke between 1–19 years prior to the interview, potentially affecting the reliability of their memories relating to the stroke, and this could also bias the findings (Sandelowski, 1993). However, this threat to the validity may have been mitigated by the fact that the study questions were focused on the informants’ current lived experience.

Future research could include an examination of the co-creation of resilience which would expand the concept beyond individual adjustment to encompass a partnership between individuals/family caregivers and broader developments at service system and societal levels, creating a more responsive milieu for people with stroke. This idea of a co-creation of resilience has been applied to a range of diverse areas such as urban planning (Labaka et al., 2019), ecology (Kench et al., 2018) and curriculum development in education (Morote et al., 2020), and could be applicable to the domain of health, including stroke. In addition, greater access to initiatives including self-management programmes (Fryer et al., 2016; Warner et al., 2015) and telehealth rehabilitation (Sarfo et al., 2018)could address a number of the service system barriers to strengthen resilience and improve quality of life. More specifically, existing interventions that build activities that have a value for patients after stroke could be investigated, to see whether they do contribute to increased resilience, as well as interventions that specifically target the building of resilience after stroke. For example, qualitative evaluation of a positive mental health programme found that it had potential to foster resilience and help overcome anxiety and depression after stroke (Mavaddat et al., 2017). Furthermore, future studies could also investigate whether there are gender differences in the how resilience is manifested.

## Conclusion

This study investigated the lived experience of QoL, participation and resilience and found that life with stroke has been adapted to but not accepted. Important factors for adaptation is meaningful values in life, individual strategies for adaptation and support from both social relationship and the society.

## Appendix 1

**Interview guide**

*Quality of life*:

What is quality of life for you?

How do you experience your quality of life today compared to before the injury?

What are your hobbies?

What were your hobbies before the injury?

Do you have any support to be able to pursue hobbies, in which case, what support?

*Participation and treatment*:

How do you experience your participation today compared to before the injury? (association life, relationships, leisure, social contexts)

What support do you have today to increase your participation?

How do you feel that accessibility works for you to be able to participate? (Physical availability, information availability, communication availability)

Do you use any aids in your everyday life? Which? (walker, wheelchair, work chair for the kitchen other)

Do you use your aids?

How do you feel that people around you treat you today compared to before the injury?

How do you feel it works with social relationships today compared to before the injury? (From friends, from your close relatives?)

*Resilience*

How important have the following factors been when you think about your “recovery journey” after the stroke?

(i) That you have the support you have needed from others.

(ii) That you have found strength or meaning in life from spirituality.

(iii) That you have an ability not to give up when things get difficult but to keep trying to achieve the goals you have set.

(iv) That you can solve the problems you face.

(v) That you have hope for the future.
